# Characterization of the duck enteritis virus UL55 protein

**DOI:** 10.1186/1743-422X-8-256

**Published:** 2011-05-24

**Authors:** Ying Wu, Anchun Cheng, Mingshu Wang, Shunchuan Zhang, Dekang Zhu, Renyong Jia, Qihui Luo, Zhengli Chen, Xiaoyue Chen

**Affiliations:** 1 Institute of Preventive Veterinary Medicine, Sichuan Agricultural University, Wenjiang, Chengdu city, Sichuan, 611130, P.R.China; 2 Avian Disease Research Center, College of Veterinary Medicine of Sichuan Agricultural University, 46 Xinkang Road, Ya'an, Sichuan 625014, P.R. China; 3 Key Laboratory of Animal Disease and Human Health of Sichuan Province, Sichuan Agricultural University, Wenjiang, Chengdu city, Sichuan, 611130, P.R.China

## Abstract

**Background:**

Characteration of the newly identified duck enteritis virus UL55 gene product has not been reported yet. Knowledge of the protein UL55 can provide useful insights about its function.

**Results:**

The newly identified duck enteritis virus UL55 gene was about 561 bp, it was amplified and digested for construction of a recombinant plasmid pET32a(+)/UL55 for expression in Escherichia coli. SDS-PAGE analysis revealed the recombinant protein UL55(pUL55) was overexpressed in Escherichia coli BL21 host cells after induction by 0.2 mM IPTG at 37°C for 4 h and aggregated as inclusion bodies. The denatured protein about 40 KDa named pUL55 was purified by washing five times, and used to immune rabbits for preparation of polyclonal antibody. The prepared polyclonal antibody against pUL55 was detected and determined by Agar immundiffusion and Neutralization test. The results of Wstern blotting assay and intracellular analysis revealed that pUL55 was expressed most abundantly during the late phase of replication and mainly distributed in cytoplasm in duck enteritis virus infected cells.

**Conclusions:**

In this study, the duck enteritis virus UL55 protein was successfully expressed in prokaryotic expression system. Besides, we have prepared the polyclonal antibody against recombinant prtein UL55, and characterized some properties of the duck enteritis virus UL55 protein for the first time. The research will be useful for further functional analysis of this gene.

## Background

Duck enteritis virus (DEV), alternatively known as Duck plague virus (DPV), is a fatal pathogen of the family Anatidae of the order anseriformes[1], leading to an acute, febrile, contagious, and septic disease to waterfowls of all ages. The resulting disease designated as duck virus enteritis (DVE) has caused serious losses in commercial duck production in domestic and wild waterfowl since it was firstly discovered in Netherlands[2]. To our knowledge, DEV has been clustered to the subfamily of alphaherpesvirinae according to the report of the Eighth International Committee on Taxonomy of Viruses (ICTV)[3]. However, it has not been classified to any genus yet.

The genome of DEV is composed of a linear, double stranded DNA. In recent years, due to the advent of molecular biology and advancements in research related to it, a lot of DEV genes has been identified, such as US2 to US5 and US10 genes[4,5], UL6 and UL7 genes[6], UL10[7], UL24, TK and gH genes[8], UL27[9], UL31[10], UL35[11], UL38[12], UL45 to UL47 [13-15], UL51[16], gK[17], gC[18], gE[19], gI[20] and so on. Even so, a great deal of unkown DEV genes remain to be clarified to facilitate the investigation of this virus. DEV UL55 gene [GenBank:EU071034] was a kind of that gene whose properties and functions has not been elucidated yet since it was identified in our laboratory in 2006[21]. To our best knowledge, the UL55 gene of alphaherpesviridae was supposed to be a late gene. Reports about HSV-2 UL55 protein revealed that the product of HSV-2 UL55 gene may play an accessory role in virion assembly or maturation[22], but the corresponding homologue gene of EHV-1 was supposed to mediate persistent infection[23]. However, the characterization of the DEV UL55 protein (pUL55) remains unclear.

To investigate the characteristics of DEV pUL55, we amplified the DEV UL55 gene by PCR and constructed a recombinant plamid pET32a(+)/UL55 for prokaryotic expression. The expression of recombinant pUL55 was induced by the addition of isopropyl-β-D-thiogalactopyranoside (IPTG) and supposed to be maximum after optimization. Polyclonal antibody was prepared by immune rabbits with purified pUL55, and then detected by agar immunodiffusion and viral neutralization test. It was subsequently used to determine the expression and subcellular localization of pUL55 in DEV infected cells. This work was supposed to facilitate the understanding of DEV pUL55 and its functional location in infected cells.

## Methods

### Computer analysis of DEV pUL55

A complete ORF of DEV CHv strain was first identified in our laboratory[21] and designated as UL55 gene. It was about 561 bp and expected to encode a protein comprising 186 amino acids with a putative molecular mass of 20.7981 KDa. A series of bioinformatics aided tools were used to analyze the intracelluar location of pUL55 : PSORT II Prediction(from the website http://psort.nibb.ac.jp/form2.html)[24], TargetP 1.1 (from the website http://www.cbs.dtu.dk/services/TargetP/)[25], SignalP 3.0(from the website http://www.cbs.dtu.dk/services/SignalP)[26], TMHMM 2.0 server (from the website http://www.cbs.dtu.dk/services/)[27], PredictNLS server(from the website http://www.rostlab.org/services/predictNLS/)[28], CSS-Palm 2.0 online server (from the website http://csspalm.biocuckoo.org/online.php)[29], and the Golgi predictor (from the website http://ccb.imb.uq.edu.au/golgi/golgi_predictor.shtml)[30]. Prediction of them were based on the putative amino acid sequence of pUL55.

### Cells, viruses, serums, and vectors

Duck embryo fibroblasts (DEF) were cultured in modified eagle's medium (MEM)(Gibco-BRL) supplemented with 10% fetal bovine serum (FBS)(Gibco-BRL), 100 U/ml penicillin, and 100 μg/ml streptomycin at 37°C. MEM medium supplemented with 2-3% FBS was used for virus infection[31]. DEV CHv strain and rabbit anti-DEV serum were obtained from Key Laboratory of Animal Disease and Human Health of Sichuan Province[32,33]. Besides, Escherichia coli strain DH5α, Escherichia coli BL21 (DE3) and expression vector pET-32a(+) were preserved in our laboratory.

### Expression and purification of recombinant UL55 protein

The amplified DEV UL55 gene was directionally cloned to pMD18T as previously discribed[34]. After confirmation by sequencing, the digested gene fragment of the recombinant plasmid pMD18-T/UL55 (retrieving by TIANgel Midi purification Kit) was directionally ligated into the previously *BamH I*/*Xho I-*digested expression vector pET32a(+), gernerating a recombinant plasmid pET32a(+)/UL55. Subsequently, the PCR, restriction enzyme digestion and DNA sequencing (TaKaRa) tests were performed to ensure the correct insertion. After that, the positive recombinant plasmids were transformed to Escherichia coli BL21 (DE3) for expression by the addition of isopropyl-β-D-thiogalactopyranoside (IPTG). The tempreture and duration of IPTG and its working concentration were optimized as descried to maximize the expression of pUL55[10]. Cells were centrifugated and lysed in 5×sample buffer (0.1 M Tris-HCl, 4% SDS, 0.2% bromophenol blue, 20% glycerol, and 0.1 M DTT, pH 6.8), then analyzed by SDS-PAGE. The uninduced control culture and the vector control culture were analyzed in parallel.

The recombinant pUL55 was purified under denaturing condition by repeated washing[35]. The induced cells were centrifugated at 10,000 rpm/min for 10 min, and resuspended in 20 mM Tris buffer (pH 8.0) with the addition of 0.1 mg/ml lysozyme (0.1 mg/ml) at -20°C overnight. The cell lysate was then sonicated on ice for 5 min at an amplitude of 30% with a 30 s pulse frequency. After 10 min centrifugation at 10,000 rpm/min, the supernatant (soluble fraction) and pellets (insoluble fraction) of it were collected respectively for SDS-PAGE analysis. Result demonstrated that the recombinant pUL55 has formed inclusion bodies (IB). The pellets were resuspended in 20 ml washing buffer (2 M urea, 50 mM Tris-HCl buffer, 1 mM EDTA, 150 mM NaCl and 0.1% Triton X-100, pH 8.0) under constant stirring for 10 min, then followed by centrifugation at 10,000 rpm/min for 10 min at 4°C. The above steps were repeated five times to release the trapped protein. The suspension was finally centrifuged at 10,000 rpm/min for 10 min at 4°C, and resuspended in denaturing buffer containing 8 M urea, 10 mM PBS, 50 mM Tris-HCl, 50 mM NaCl, 10% glycerine, pH 8.0. The purity of pUL55 was tested by SDS-PAGE.

### Western blotting assays[12]

Western blotting assay was performed using the purified rabbit anti-DEV (diluted 1:200) IgG to characterize the reactivity and specificity of the recombinant pUL55. The purified recombinant pUL55 were separated by 12% SDS-PAGE and transferred onto polyvinylidene fluoride (PVDF) membrane at 120 V for 1.5 h in a BioRad mini Trans-Blot electrophoretic transfer cell (BioRad, Shanghai, China). Blocking the membrane with 10% skimmed milk in TBST (Tris-buffered saline with 0.1% Tween-20, pH 8.0) for 1 h at 37°C or overnight at 4°C. Sequently, the membrane was incubated with appropriate dilution of rabbit anti-DEV (diluted 1:200) serum for 1 h at 4°C overnight. After washing three times, the HRP-conjugated goat anti-rabbit IgG (diluted 1:3000) was added for incubation. Pre-serum came from non-immune healthy rabbit blood was disposed parallelly for control. One hour later, washing the membrane with TBST as before, followed by 3 min for color development with substrate solution (DAB 3'3'-Diaminobenzidine tetrahydrochloride peroxidase) at 37°C. The reaction was terminated by thoroughly washing with distilled water.

### Preparation of polyclonal antibody against recombinant pUL55

Renaturation of recombinant pUL55 was carreied out by dilution method and gradient dialysis[31]. Firstly, the refolding buffer (1 mM EDTA, 0.15 M NaCl, 50 mM Tris-HCl, 1 mM GSSG, 1 mM GSH and 1% Arginine, pH 8.0) was added to the denatured pUL55 slowly until the urea concentration reached 6 M. Sequently, the partly refolded protein was dialyzed in different concentrations of urea buffer solution (6 M, 4 M, 3 M, 2 M) containing 50 mM Tris-HCl, 50 mM NaCl, 0.5 mM EDTA and 10% glycerine, pH 8.0 at 4°C. Changing the dialyzate of each at least three times a day. At last, the aggregation was removed by centrifugation and the supernatant was collected as soluble refolded protein.

For preparation of polyclonal antibodies[31], male New Zealand white rabbits were first immunized intradermally with a mixture of 0.5 mg renatured recombinant pUL55 and an equal amount of complete Freund's adjuvant (Sigma, Shanghai, China). Two weeks later, 0.75 mg purified fusion pUL55 and an equal amount of Freund's incomplete adjuvant were used for secondary immunity. After that, the rabbits were boosted subcutaneously with 1.0 mg each of recombinant pUL55 and an equal amount of incomplete Freund's adjuvant at a 1-week interval. Seven days later, the rabbits were injected intravenously with 0.1 mg purified pUL55 each. At last, serums were collected 17 days later. Control pre-immune serum was obtained from the non-vaccinated healthy rabbits.

The obtained rabbit polyclonal anti-serum against pUL55 was subsequently purified by ammonium sulfate precipitation and High-Q anion-exchange chromatography[36] following the manufacturer's instructions. The purified IgG fraction was analyzed by 12% SDS-PAGE.

### Agar diffusion reaction

Agar diffusion reaction was used to detect the reactivity and specificity of the purified UL55 anti-serum[37]. One gram of agar was dissolved in 100 ml normal saline for the test. It was heated, cooled down to 55°C, and then poured into the plates to a thickness of 2 mm. After subsequent solidification with cooling, the agar was perforated with 3 mm diameter holes that may hold approximately 100 μl of solution. Twenty microliters each of the pre-immune serum, 1:2, 1:4, 1:8, 1:16 and 1:32 diluted anti-serum was added into the peripheral apertures. At last, 20 μl purified pUL55 was added into the central aperture. The plate was incubated at 37°C for 24 h before observation.

### Viral neutralization test

Viral neutralization test was used to determine the neutralizing viral antibody titer of the obtained anti-serum. DEFs were prepared as we described above, and 350 μl of cell suspension was added to each well of the 48-well plate for incubation. Sequently, inactivated anti-pUL55 serums (56°C for 30 min) were serially diluted twofold from 1:1 to 1:32. Mixing 25 μl of the 200 TCID50 (TCID50 = 10^-6.334^) virus which was diluted from the virus stock suspension previously with an equal volume of serum dilution, and incubating it at 37°C for 1 h. When the cells grew into a monolayer, 50 μl of each incubated antiserum was inoculated onto the cells for infection. Meanwhile, seven contrast controls were set up for later observation: blank control 1:2, diluted anti-serum, 200 TCID50, 100 TCID50, 10 TCID50, 1 TCID50 and 0.1 TCID50 was respectively added to the cell culture. Each dilution of these invovled serums and viruses were tested in triplicate. After 1 h adsorption at 37°C, the cells were overlaid with the MEM maintenance media for incubation. Observation the cytopathic effect (CPE) of them timely.

### The dynamic expression of UL55 protein in DEV-infected cells

DEFs infected and mock infected with DEV were harvested at 8 h, 12 h, 24 h, 36 h, 48 h, 60 h and 72 h post-infection to determine the kinetics of pUL55 expression. Cells lysate were mixed with 5×SDS sample buffer and heated at 100°C for 10 min. Then centrifugalization it before SDS-PAGE. After gel separation, proteins were transformed onto PVDF membrane for western blotting. It's worth noting that, here purified DEV UL55 IgG (diluted 1:64) substitued DEV IgG for dynamic expession analysis.

### Intracellular localization of UL55 protein in DEV-infected cells

Indrect immunofluorescent microscopy was used to investigate the intracellular location of pUL55 in infected cells[25]. Preparing monolayers of DEFs as a matter of routine, cells were expected to grow on coverslips in six-well plates and they were supposed to be mock infected or infected with DEV. At different times (5.5 h, 11 h, 16 h, 22.5 h, 30 h, 35 h, 40 h, 45.5 h, 49 h, 54 h, 60 h, 70 h and 74 h post infection), cells were harvested and fixed with 4% paraformaldehyde overnight at 4°C. Sequently, they were washed with PBS buffer and permeabilized with 0.1% Triton X-100 for 30 min. After that, washing the cells with PBS contaning 0.1% tween-20 for three times before they were blocked with PBS containing 4% BSA for at least 1 h at 37°C. Then, the cells were incubated overnight with purified UL55 IgG (1:64 diluted) in PBS containing 1% BSA at 4°C. Three times washing had been performed as decribed above before they were treated with 1:100 diluted FITC conjugated goat anti-rabbit IgG (Sino American Biotechnology Co.) at 37°C for 1 h. The cell nuclei were visualized by 4', 6-diamidino-2-phenylindole (DAPI) counter-staining (5.0 μg/ml, Beyotime Institute of Biotechnology, Shanghai, China) after washing three times. The images were captured with fluorescence microscopy (Nikon, Japan)[25].

## Results

### Prediction of subcellular localization of DEV pUL55

Computer analysis of the subcellular localization of DEV pUL55 suggested that the pUL55 was mainly located in cytoplasmic (60.9%) of infected cells, then in cytoskeletal (17.4%), nuclear (13.0%), peroxisomal (4.3%) and mitochondria (4.3%) sequentially. However, according to the prediction, DEV pUL55 contained no potential mitochondrial targeting peptide, N-terminal signal peptides, transmembrane region and nuclear localization signal (NLS). Further, Golgi prediction results indicated pUL55 was not a Golgi type II membrane protein(Golgi localised transmembrane protein) since the index values of a Golgi protein should be geater than the threshold (20.005) while the index values of pUL55 was 0.

### Expression and purification of UL55 recombinant protein

**Figure 1:**
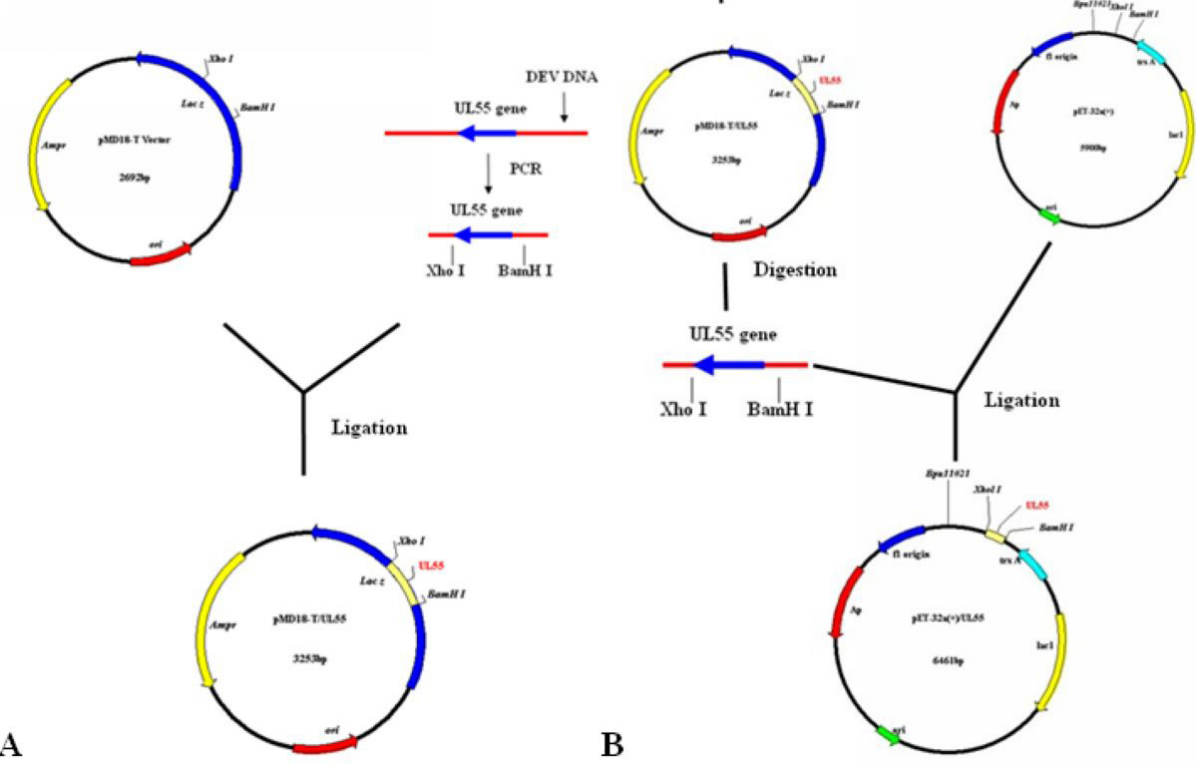
**The cloning strategy for constructing the UL55 recombinant plasmids**. **A**. Schematic diagram of the UL55 ORF cloned into the pMD18-T cloning vector. **B**. Construction of the recombinant expression plasmid pET-32a(+)/UL55.

**Figure 2:**
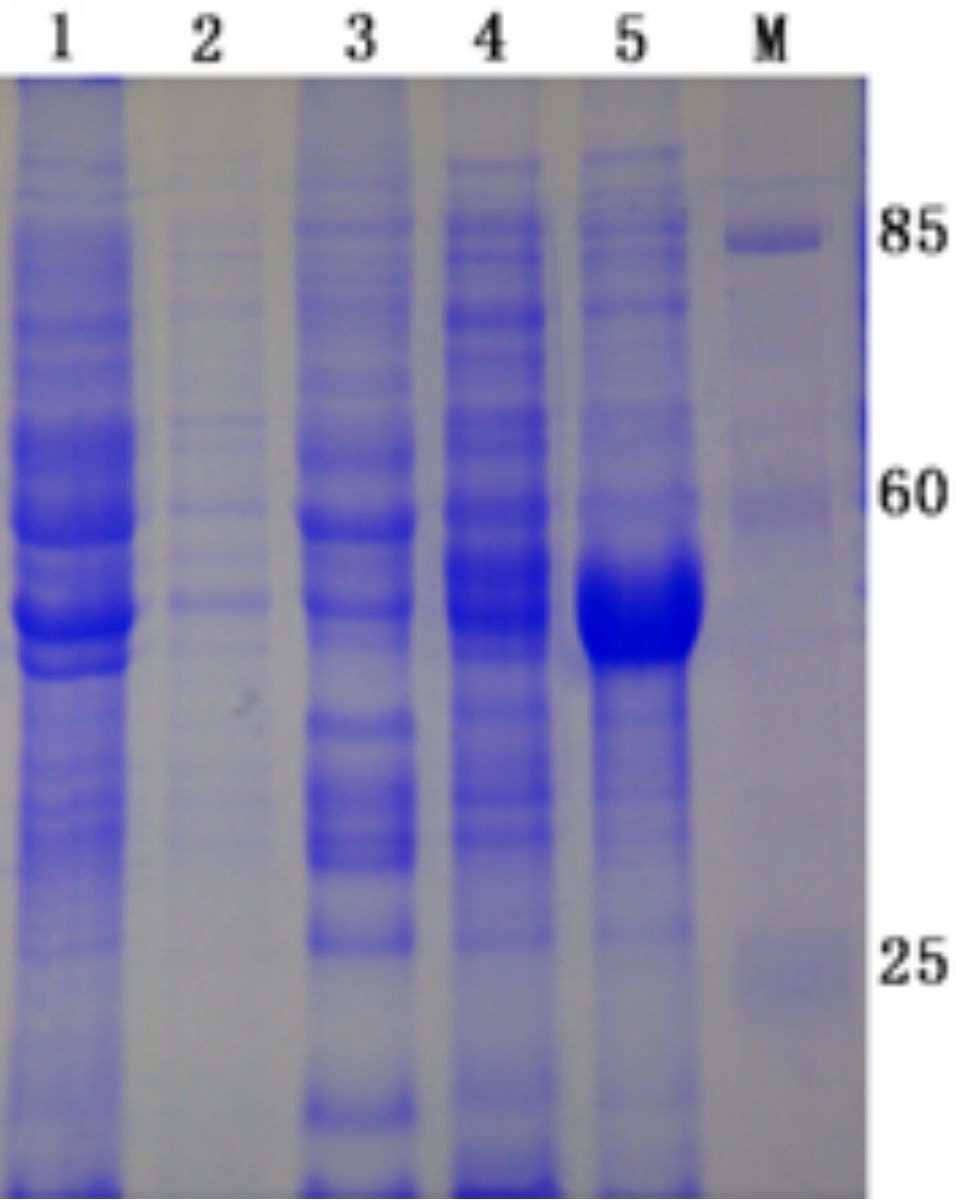
**The expression pET32a(+)/UL55 protein produced in E. coli strain BL21 (DE3)**. The pET32a(+)/UL55 protein was expressed in E. coli BL21(DE3) host strains. M represented standard protein molecular weight markers. Lane 1, the culture of pET-32a(+) after induction in E.coli BL21; Lane 2 and Lane3, the supernatant and pellet of the recombinant pET-32a(+)/UL55 cultures before induction in E.coli BL21, respectively. Lane 4 and Lane 5, the supernatant and pellet of the recombinant pET-32a(+)/UL55 cultures after induction in E.coli BL21, respectively.

**Figure 3:**
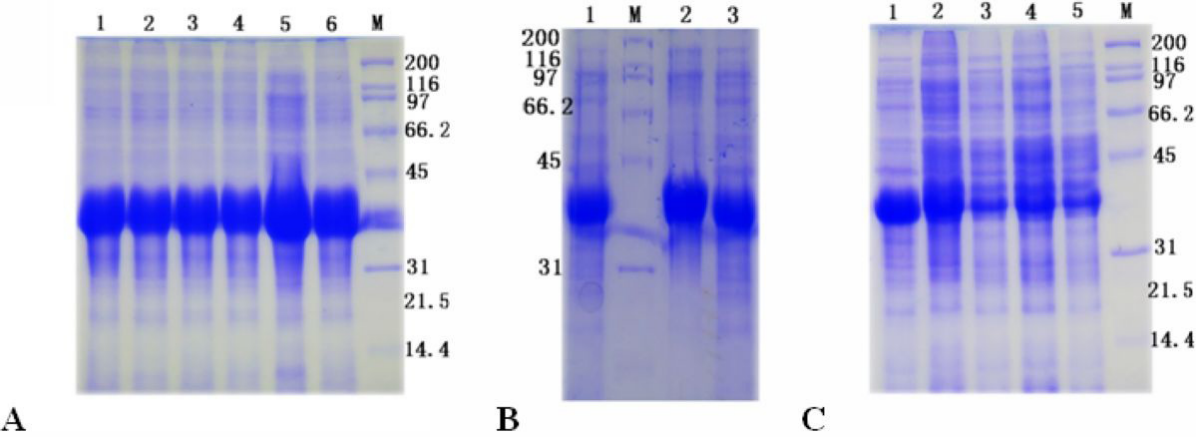
**The optimal expression condition of pET32a(+)/UL55 protein**. **A**. The determination of the optimal final concentration of IPTG. Lanes 1, 2, 3, 4, 5 and 6 represented the final concentrations of IPTG were 1.0 mM, 0.8 mM, 0.6 mM, 0.4 mM, 0.2 mM and 0.1 mM respectively. **B**. The determination of the optimal induced temperature. Lanes 1, 2 and 3 represented the induction temperatures were 30°C, 37°C and 25°C, respectively. **C**. The determination of the optimal duration of IPTG. Lanes 1, 2, 3, 4 and 5 represented the durations of IPTG were 4.0 h, 5.0 h, 6.0 h, 7.0 h and 8.0 h, respectively.

Recombinant plasmids containing the encoding region of DEV UL55 were constructed for expression. Schematic diagrams of the cloning strategy of DEV UL55 were shown in Figure 1. The constructed recombinant plasmids pET32a(+)/UL55 was transformed into E. coli BL21 (DE3) for expression. After incubation at 37°C, the cultures were analyzed by SDS-PAGE. Results demonstrated that the E. coli BL21 (DE3) transformed with recombinant plasmid pET32a(+)/UL55 expressed a considerable amounts of a 40 KDa protein and it was mainly in the insoluble fraction(Figure 2, Lane 5). However, the corresponding band of pUL55 was absent in the inducing culture of pET32a(+) vector (Figure 2, Lane 1), the cultures of pET-32a(+)/UL55 before induction (Figure 2, Lane 2-3), and the supernatant of the culture of pET-32a(+)/UL55 after induction (Figure 2, Lane 4). Figure 3 indicated the optimal expression conditions of pUL55 in E. coli BL21 containing the working concentration of IPTG for inducing, the induction tempreture and the duration of IPTG. As a result, the maximum expression of pUL55 in prokaryotic system was induced by 0.2 mM IPTG (Figure 3A, Lane 5) at 37°C (Figure 3B, Lane 2) for 4.0 h (Figure 3C, Lane 1).

**Figure 4:**
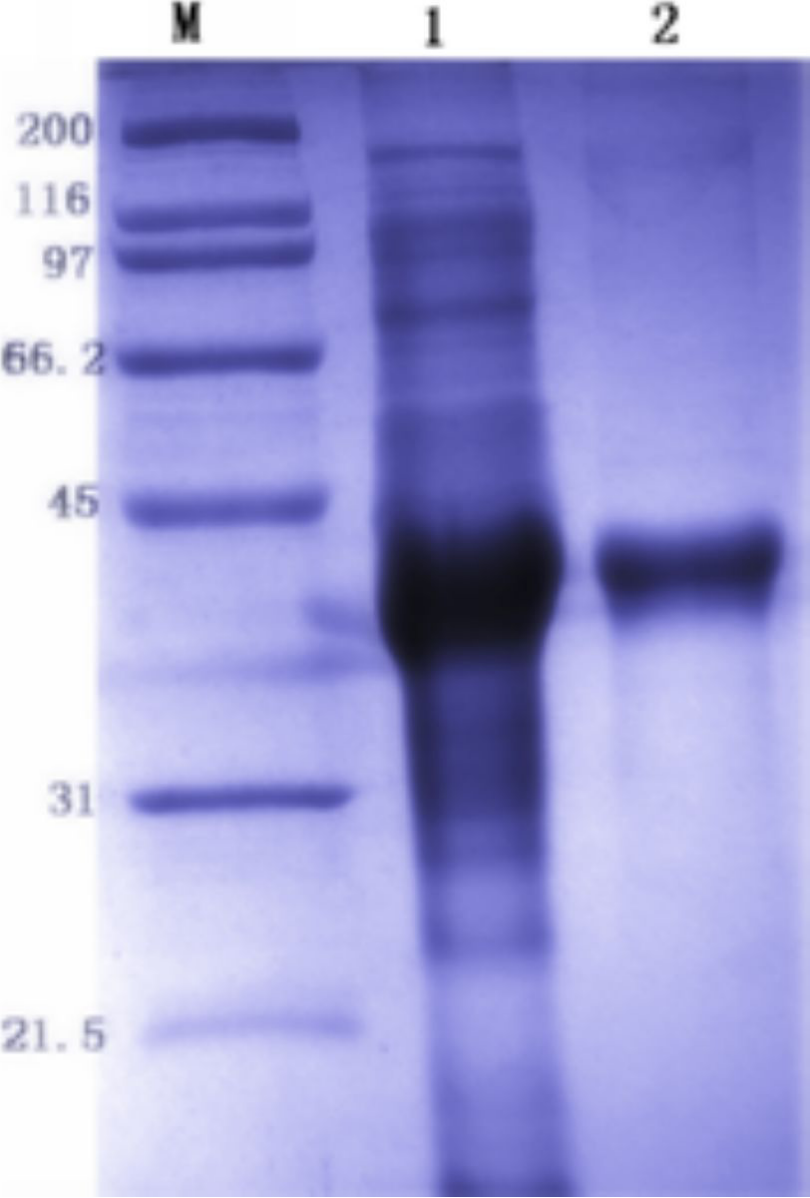
**Purification of pET32a(+)/UL55 IB**. SDS-PAGE analysis of the purity of recombinant pUL55. M represented standard protein molecular weight markers. Lane 1, the pellet of expressed pUL55 after induction in E.coli BL21; Lane 2, the expressed pUL55 was purified by washing five times.

**Figure 5:**
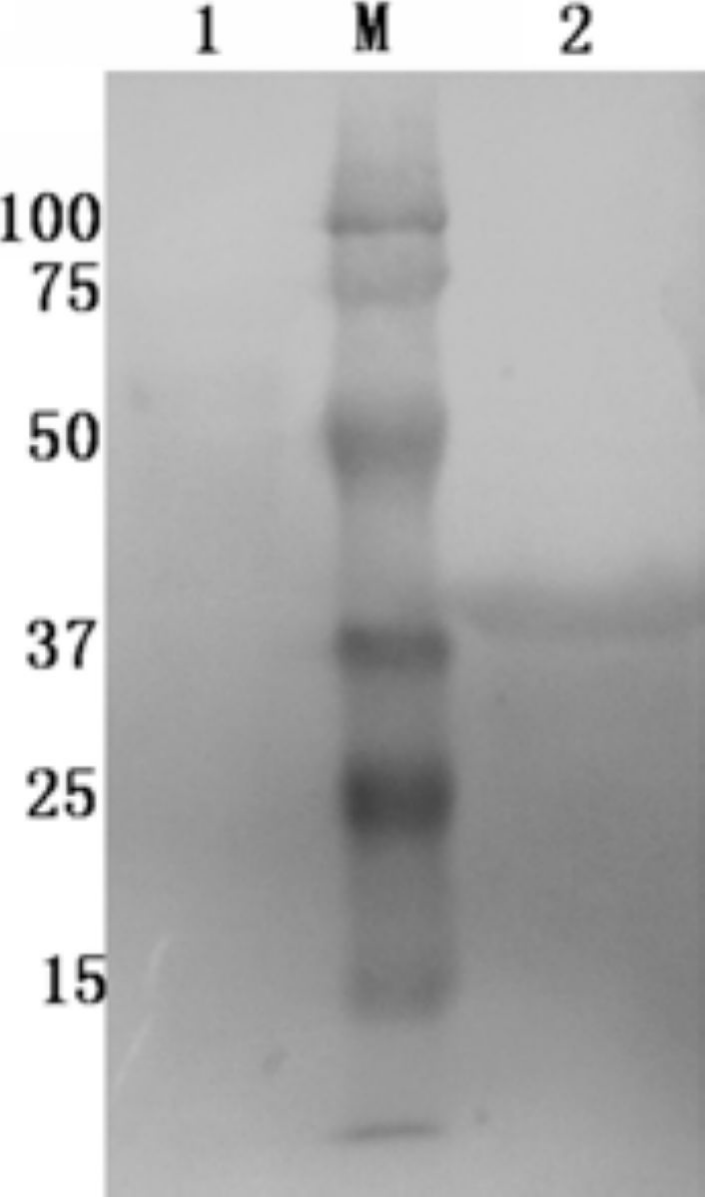
**The immunoreactivity of the recombinant UL55 protein**. The immunoreactivity of the recombinant UL55 protein was analyzed by western blotting assay with the rabbit anti-DEV IgG. M: Prestained Protein Molecular Weight marker; Lane 1, the purified pUL55 was analyzed with pre-serum. Lane 2, the purified pUL55 was analyzed with the rabbit anti-DEV monoclonal antibody.

Purification of DEV pUL55 was performed under denaturing condition since Figure 2 has demonstrated most of the pUL55 were expressed as insoluble inclusion bodies (IB) in E. coli. Eluant containing 2 M urea was used for purification. After washing five times, the purified pUL55 was dissolved finally in 8 M urea. SDS-PAGE analysis demonstrated the purity of pUL55 after washing (Figure 4, Lane 2) was higher compared to the crude pUL55 (Figure 4, Lane 1). Immunogenicity of the purified pUL55 was detected by Western blotting assay. As shown in Figure 5, the DEV anti-serum can specifically recognized a 40 KDa band (Figure 5, Lane 2), which corresponded to the theoretical molecular mass of pET32a(+)/UL55. However, no positive signal was observed when using the pre-immune serum in western blotting (Figure 5, Lane 1).

**Figure 6:**
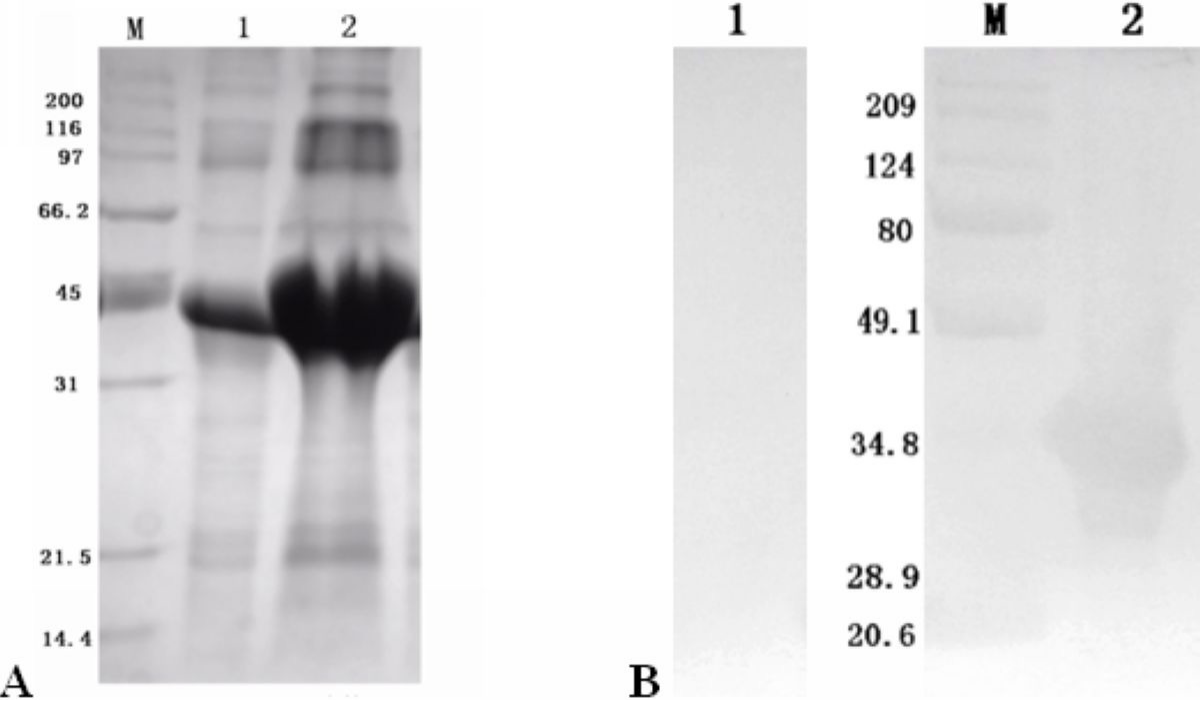
**Identification of the renatured pET32a(+)/UL55 protein**. The renaturation of pUL55 was detected by SDS-PAGE and Western blotting assay using anti-DEV serum for recognization. **A**. M: Molecular mass marker; Lane 1, the renatured pUL55; Lane 2, the recombinant pUL55 without purification and renaturation. **B**. M: Prestained Protein Molecular Weight Marker; Lane 1, identified the renatured pUL55 with pre-serum; Lane 2, identified the the renatured pUL55 with the rabbit anti-DEV IgG.

Purified pUL55 was supposed to be refolded by dilution method and gradient dialysis. SDS-PAGE was performed to analysis the renatured pUL55 (Figure 6A, Lane 1) firstly. Then anti-DEV serum was used to recognize the renatured pUL55. Band about 40 KDa represented pUL55 was observed by Western-blotting assay (Figure 6B, Lane 2), indicating that the renatured pUL55 reacted with anti-DEV serum. Corresponding band was absent when recognized by pre-immune serum (Figure 6B, Lane1).

### Verification the character of polyclonal antibody against DEV pUL55

**Figure 7:**
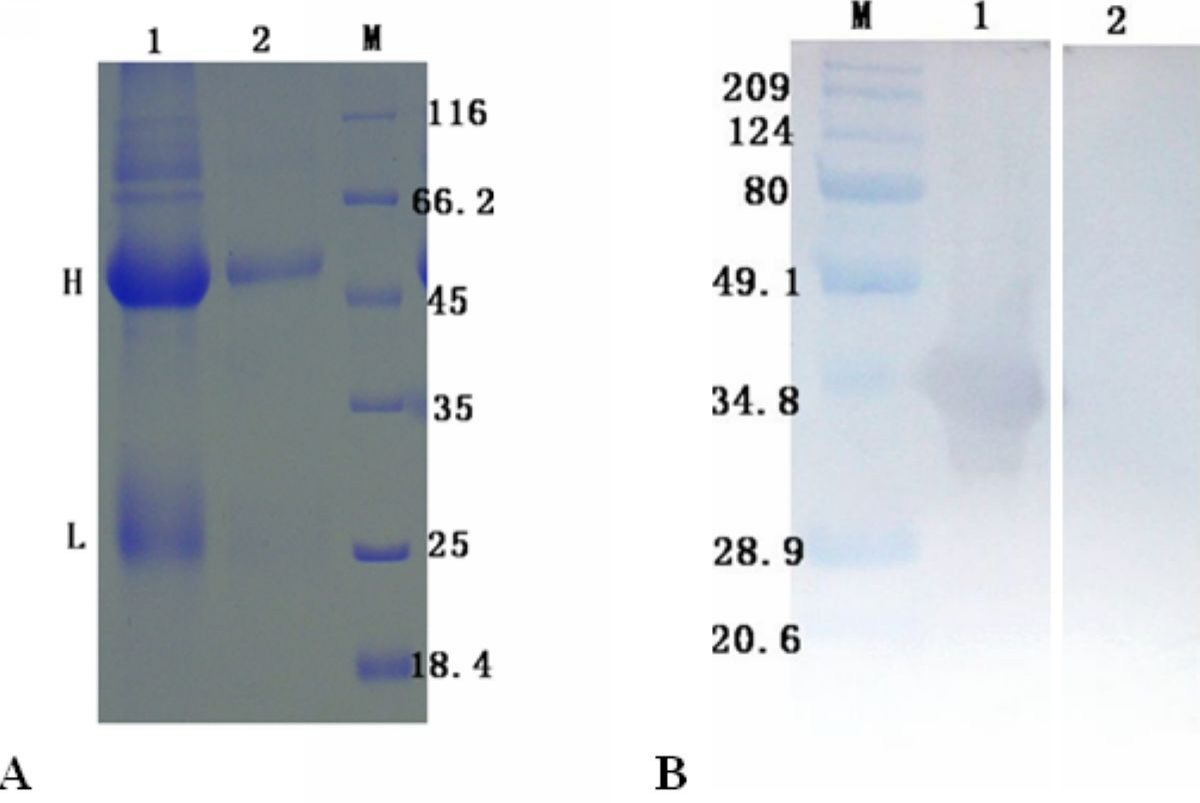
**Verification the character of the UL55 polyclonal antibody**. **A**. SDS-PAGE analysis of the anti-pUL55 serum and purified anti-pUL55 serum. M: Molecular mass marker; Lane 1, The IgG proteins of UL55 antiserum examined by SDS-PAGE; Lane 2, The purified IgG of pUL55 examined by SDS-PAGE. H and L indicated the position of heavy and light chains of the IgG proteins, respectively. **B**. The renatured pUL55 was recognized by purified anti-pUL55 serum. M: Prestained Protein Molecular Weight Marker; Lane 1, the renaturated UL55 protein was analyzed with purified anti-pUL55 IgG; Lane 2, the renaturated UL55 protein was recognized by pre-serum.

Polyclonal antibodies against DEV pUL55 obtained from immune rabbits were purified before using. SDS-PAGE analysis described the purification result of anti-pUL55 serum (Figure 7A, Lane 2) by comparison. The reactivity and specificity of it was detected by Western blotting assay. As shown in Figure 7B, the purified anti-pUL55 serum reacted strongly with an approximate 40 KDa protein which represented renatured DEV pUL55 (Figure 7B, Lane 1). However, the corresponding band was not found when using pre-immune serum (Figure 7B, Lane 2).

**Figure 8:**
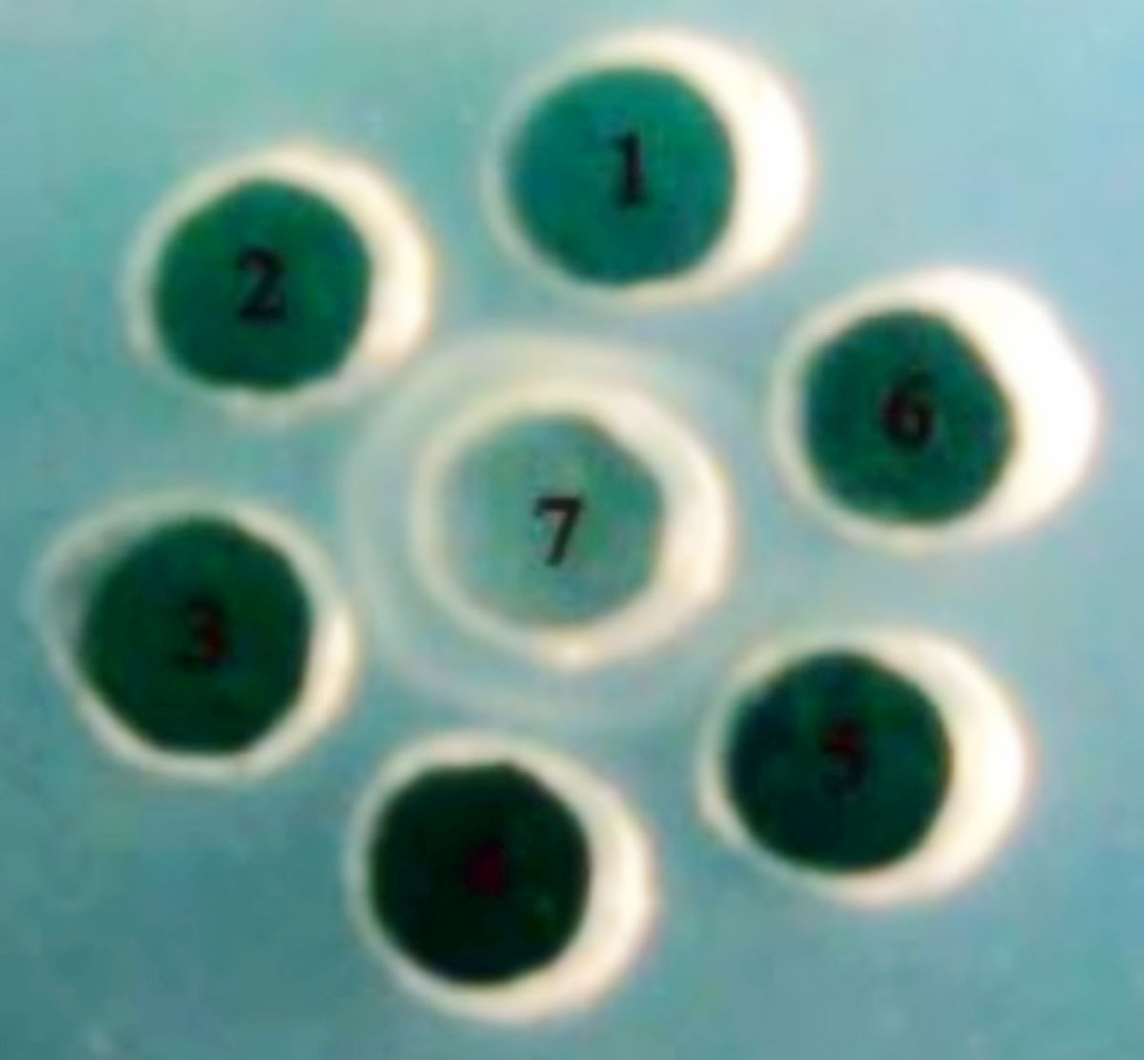
**Agar diffusion reaction**. Agar diffusion reaction was designed for dectecting specificity and sensitivity of anti-pUL55 serum. The result of agar diffusion reaction of the anti-pUL55 serum with purified pUL55 suggested the largest dilution multiple of it was 1:16. 1-5, represented 1:2, 1:4, 1:8, 1:16 and 1:32 diluted anti-seraum, respectively; 6, rabbit pre-immune serum; 7, Purified pUL55.

Agar diffusion reaction was performed to determine the immunoreactivity of anti-pUL55 serum with purified pUL55. Figure 8 suggested the highest titer of the agar diffusion reaction of anti-pUL55 serum with pUL55 was 1:16 (Figure 8, lable 4). Pre-immune serum used as a negative control didn't show any antigen-antibody complexes (Figure 8, lable 6).

Observation of the neutralization titer of the rabbit anti-pUL55 polyclonal antibody was detected by micro neutralization test. Calcutating 50% serum neutralized through Reed-muench method[38]. As a result, the neutralization titer of the rabbit anti-UL55 polyclonal antibody was 1:7.484 (data not shown).

### Dynamic expression of pUL55 in DEV-infected cells

**Figure 9:**
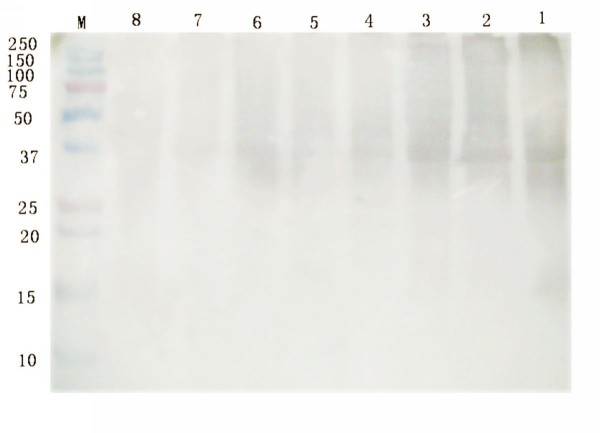
**Dynamic expression of UL55 in DEV-infected cells**. The kinetics of pUL55 was examined by SDS-PAGE and Western blotting assay. M: Prestained Protein Molecular Weight Marker; Lane 1-7, DEFs infected with DEV were harvested at 8 h, 12 h, 24 h, 36 h, 48 h, 60 h and 72 h post-infection. The lysates of DEV-infected was analyzed by rabbit anti-pUL55 serum. Lane 8, DEFs mock infected with DEV were done in parallel.

DEFs mock-infected or infected with DEV were analyzed by western blotting assays at a series of time post infecion for the purpose of monitoring the dynamic expression of pUL55. Cells were harvested at different time, and separated by SDS-PAGE. Then, proteins were electrophoretically transferred onto PVDF membrane for Western blotting analysis using anti-pUL55 serum. Result in Figure 9 revealed that the DEV pUL55 was easily detected as early as 8 h p.i (Figure 9, Lane 1) and seemed to keep increasing until maximum at 24 h p.i (Figure 9, Lane 3), after that, a visble band was present at decreased levels untile 60 h p.i (Figure 9, Lane 1).

### Intracellular localization and distribution of DEV pUL55 in DEV-infected cells

**Figure 10:**
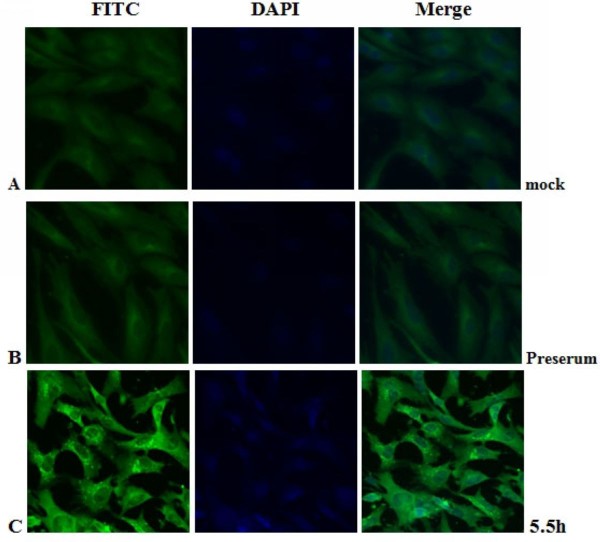
**Intracellular localization of DEV pUL55 in mock-infected cells and DEV-infected cells at 5.5 h post-infection**. **A**. Mock-infected cells were fixed; **B-C**. DEV-infected cells were fixed at 5.5 h post-infection; The sample B was incubated with rabbit pre-immune serum while samples A and C were incubated with anti-pUL55 serum and subsequently stained with fluorescein isothiocyanate(FITC)-conjugated secondary antibody. Nuclei were counterstained with DAPI(blue). The merged fluorescence microscopy image of DEF are shown in panels A-C with high magnification (600×).

**Figure 11:**
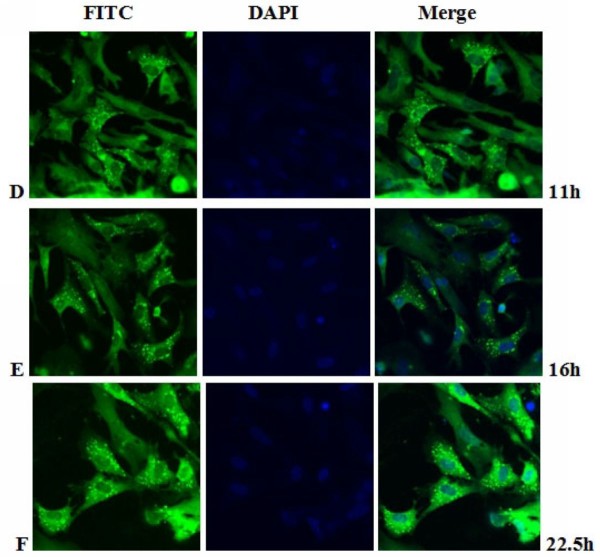
**Intracellular localization of DEV pUL55 in DEV-infected cells at 11 h, 16 h, 22.5 h post-infection**. **D-F**. DEV-infected cells were fixed at 11 h, 16 h, 22.5 h post-infection. Samples were incubated with anti-pUL55 serum and subsequently stained with fluorescein isothiocyanate(FITC)-conjugated secondary antibody. Nuclei were counterstained with DAPI(blue). The merged fluorescence microscopy image of DEF are shown in panels D-F with high magnification (600×).

**Figure 12:**
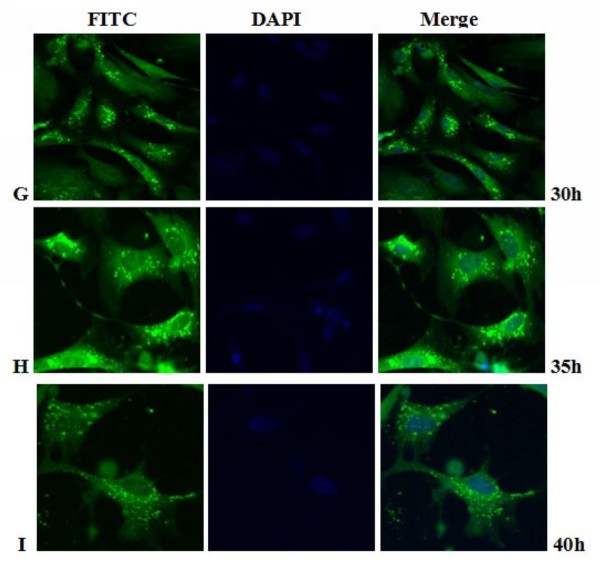
**Intracellular localization of DEV pUL55 in DEV-infected cells at 30 h, 35 h, 40 h post-infection**. **G-I**. DEV-infected cells were fixed at 30 h, 35 h, 40 h post-infection. Samples were incubated with anti-pUL55 serum and subsequently stained with fluorescein isothiocyanate(FITC)-conjugated secondary antibody. Nuclei were counterstained with DAPI(blue). The merged fluorescence microscopy image of DEF are shown in panels G-I with high magnification (600×).

**Figure 13:**
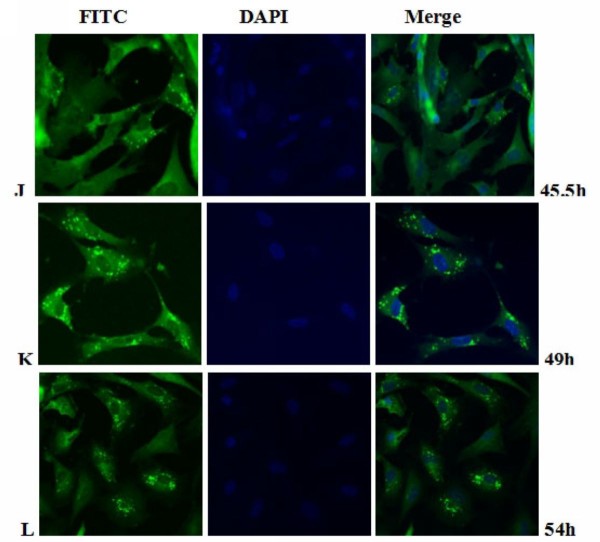
**Intracellular localization of DEV pUL55 in DEV-infected cells at 5.5 h, 49 h, 54 h post-infection**. **J-L**. DEV-infected cells were fixed at 45.5 h, 49 h, 54 h post-infection. Samples were incubated with anti-pUL55 serum and subsequently stained with fluorescein isothiocyanate(FITC)-conjugated secondary antibody. Nuclei were counterstained with DAPI(blue). The merged fluorescence microscopy image of DEF are shown in panels J-L with high magnification (600×).

**Figure 14:**
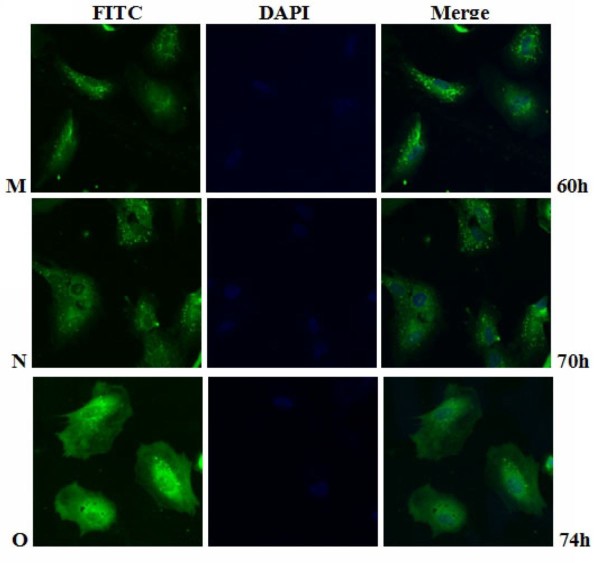
**Intracellular localization of DEV pUL55 in DEV-infected cells at 60 h, 70 h and 74 h post-infection**. **M-O**. DEV-infected cells were fixed at 60 h, 70 h and 74 h post-infection. Samples were incubated with anti-pUL55 serum and subsequently stained with fluorescein isothiocyanate(FITC)-conjugated secondary antibody. Nuclei were counterstained with DAPI(blue). The merged fluorescence microscopy image of DEF are shown in panels M-O with high magnification (600×).

The intracellular distribution of pUL55 in DEV infected cells was examined by indirect immunofluorescence staining with purified anti-pUL55 serum. At various times after infection, DEF cells were collected and fixed in cold paraformaldehyde. Optimization results revealed the coverslips were expected to be fixed at 4°C overnight with 4% cold paraformaldehyde, and then treated with 4% BSA to block the nonspecific staining, the permeabilization time was with 0.2% (v/v) TrionX-100 in PBS for an additional 30 min at 4°C and the anti-pUL55 IgG was supposed to diluted 1:64 to incubate at 4°C overnight in the coverslips (datas not shown). As shown in Figure 10C, the pUL55 was distributed in bright fluorescent granules in the cytoplasm of infected cells at 5.5 h p.i. However, these fluorescence pellets were absent from mock-infected cells (Figure 10A), and no significant fluorescence was observed with the preimmune serum(Figure 10B). After that, the detectable fluoresecence structures kept increasing, the strongest fluorescence was observed at 22.5 h p.i (Figure 11F). From Figure 10C to Figure 12H, we easily found the bringht fluorescence granules were widely distributed in the cytoplasm and gradually near the periphery of the nucleus even traces of them within nuclear. Starting from 40 h p.i, the fluorescence granules expressed diffusely throughuout the cytoplasm then reclustered to bright speckled structures which distributed especially in the juxtanuclear region (from Figure 13J to Figure 14M). These fluorescence gradually diminished as time going on. Meanwhile, the DEF cells turned into the round shape and began to shed off at 54 h p.i. At 74 h post infection (Figure 14O), the pUL55-specific fluorescence almost vanish following the cytoplasm disintegration in infected cells.

## Discussion

The product of DEV UL55 gene which has been designated as pUL55, was a 186 amino acids protein encoded by a 561 bp ORF[26]. In our research, a series of experiments were preformed to characterize the duck enteritis virus UL55 protein. As the first step towards studying the characterization of the DEV pUL55, the digested UL55 fragment was directionally inserted into the pMD18-T and pET32a(+) vector sequentially to constrcut recombinant plasmids (Figure 1). PCR, Restriction enzyme digestion and DNA sequencing were used to comfirm the correctness of insertion as described previously[34]. The determined recombinant plasmid pET32a(+)/UL55 was transformed into Escherichia coli BL21 for prokaryotic expression. The optimal expression condition of recombinant pUL55 was induced by 0.2 mM IPTG at 37°C for 4 h. A 6×His-Tag fusion pUL55 approximately 40 KDa was collected as inclusion bodies in exprssion procedure and can be easily purified after washing five times under denaturing conditions. The refolded pUL55 could be recognized by rabbit anti-DEV IgG through western blotting assay which suggested a good immunogenicity of pUL55. Dilution method and gradient dialysis were used to restore the natural structure of denatured pUL55. SDS-PAGE and western blotting analysis indicated that the renatured pUL55 obtained higher purity and immunogenicity which was more suitable for producing specific polyclonal antiserum of pUL55.

The obtained rabbit polyclonal UL55 IgG in our work was purified using ammonium sulfate precipitation and High-Q anion-exchange chromatography. SDS-PAGE analysis of the extractive anti-pUL55 IgG detected two expected bands about 55 KDa and 25 KDa respectively. The refolded pUL55 was used to recognize the extractive anti-pUL55 IgG by western blotting assay. These results indicated that the renatured pUL55 has induced a strong immunological response and the prepared antiserum had a high level of specificity. It can be widely used for identification features of DEV UL55 gene product. The titer of agar diffusion reaction reached 1:16 which suggested the extractive anti-pUL55 IgG was specific and sensitive to pUL55. Moreover, the determined titers of Viral neutralization test demonstrated that pUL55 can neutralized DEV and anti-DEV infection, also has the potential to produce subunit vaccines[39].

Kinetics of UL55 expression in DEV infected DEFs was determined by western blotting. Results suggested the DEV pUL55 became detectable as early as 8 h p.i, increased in amount and reached it highest level at 24 h p.i. No appreciable protein was detected until 60 h p.i. The DEV UL55 protein existed in infedcted cells almost throughout the viral replication cycle. In the temporally regulated cascade of herpesvirus gene expression, the products of herpesvirus genes has been divided into three types according to the transcription conditions of HSV-1, PRV[40], HCMV[41]. Proteins encoded by immediate-early (IE) and early (E) genes were supposed to be expressed firstly which might be involved in virus replication. The following expressed proteins were structual proteins of virus encoded late(L) genes which were further subdivided into two categories as leaky-late (γ1) or strict-late (γ2)[18]. The last kind of proteins were some nonessential proteins encoded by optional genes. To our knowledge, the protein kinase pUS3[42] and dUT-Pase[43] wich were first detected at 2 h.p.i. and 4 h p.i. respectively has been defined as immediate-early products. By contrast, the pUL31[10] and pUL51[25] of DEV were classified to late gene products since they were first detected at 6 h.p.i. and 8 h.p.i, respectively. Consequently, the pUL55 was concluded to be the product of a late gene and might be a component of DEV virions. Researches about HSV-2 UL55 gene product in infected cells suggestted the pUL55 protein was synthesized as a γ2 gene but not a stable component of HSV-2 virions[22].

Viruses use the host synthetic machinery for replication. Viral proteins need to be targeted to the appropriate intracellular compartments of the host cell to fulfill their roles[25]. Regional distribution of protein in cells will influence the procedures of protein folding, polymn and post-transcriptional modification. Then further affect the fuctions of cell[44]. Only if the synthetic protein be transformed into specific organelle did the vital movements working orderly. Any deviation of location will have significant impacts on functions even the vital movement of cells. Proteins which merely located in nucleus are expected to participate the metabolic processes of DNA or RNA in cells. Otherwise, the proteins distributed in cytoplasm or cytolemma have nothing to do with the above procedures. Study the intracellular location of proteins will increase our understanding of the role of these proteins in host cells and could also be useful for the design of improved therapeutic interventions.

Previously research indicated that the indirect immunofluorescence experiments was a useful method for subcellular location of protein in infected cells[25]. In fact, it is a specific, sensitive and rapid antigen-antibody binding reaction. In our research, we found the location of DEV pUL55 in infected cells was dynamic changes during the life cycle of DEFs. That probably means the pUL55 has an important realationship with the propagation of DEV in DEFs. Results in Figure 10 to Figure 14 suggested the pUL55 was predominantly located in cytoplasm as the computational analysis predicted (60.9% into cytoplasmic), and small amount of it within nuclear. It started to expression in cytoplasm as early as 5.5 h p.i, then diffusion to cytoplasm and gradually distributed near the periphery of the nucleus between 11 h p.i and 35 h p.i. After that, the fluorescence granules clustered to speckled structures and distributed dominantly in the juxtanuclear region from 40 h p.i. At last, the fluorescence diminished since 54 h p.i that suggested the intracelluar location variation of pUL55 might due to the place transformation of protein synthesis and its function exertion. It was presumed that the pUL55 might be synthesized in cytoplasm initially then transformed nearby the periphery of the nucleus to implement its biologic functions. According to previous report, HSV-2 UL55 was located within and near the periphery of nucleus and abutted on and partially overlapped the capsid protein ICP35 which would coalesced VP5, VP19c at late times p.i and located at the periphery of large globular structures composed of proteins involved in DNA replication[22]. Thus, the pUL55 located nearby the perinucler space to pariticipate in the package of virus. When packaged viurs DNA which has been wrappered by ICP35 and its neucleocapsid aggregates transformed nearby[45], the synthesized pUL55 combined to it as a tegument component[46] or something. However, it might participate in package through some unkown mechanism instead of to be a component. Besides, the distribution of fluorescence transformed once again go along with the variation of DEV viron in infected cells since 35 h p.i can be explained to be related to the propagation of virus in DEFs and cytopathic mechanism. The fuloresence structures gradually diminished to shed off afterwards probably due to the maturity, egress and release of viurs according to the acceptable propagation pattern of DEV in host cells[47]. Apart from that, pUL55 became undetectable probably because it is a low abandance protein in packaged virons[46] or it is not a stable component of DEV virions[22]. Of course, the above assumptions about pUL55 and its mechanism of involving in DEV propagation need to be determined in future studies.

Electron microscopic characterization of duck plague virus[48] suggested the initial progeny virus nuecleocapsids are detectable since 12 h p.i and the mature virus was observed at 24 h p.i. The initial 6 h are latency period of DEV[49]. In our research, pUL55 was firstly detected at 5.5 h p.i which was probably produced by parental viruses since pUL55 has been designated to be a late gene according to previrously report[22] and dynamic expression of pUL55 we had investigated above. The fluorescence granules repesented pUL55 were clusterd to peak at 22.5 h p.i corresponding to the mature time of DEV and the dynamic distribution of pUL55 in cells at 24 h p.i basically. After that, fluorescence became weak gradually due to the release of mature DEV.

## Conclusions

In this work, the recombinant plasmid pET32a(+)/UL55 was constructed successfully for expression in prokaryotic system. The purified and renatured recombinant pUL55, which was recognized well with anti-DEV serum, was used for preparation of specific anti-pUL55 serum. Viral neutralization test demonstrated that the pUL55 has the potential to produce subunit vaccines, and possesses the functions of neutralizing DEV and anti-DEV infection. The determined anti-pUL55 serum was used for characterization of pUL55 by Western blotting assay and indrect immunofluorescence. As a result, we found the expression of this gene appeared at the late stage of infection in infected DEFs and pUL55 was predominantly located in cytoplasm and traces of it in nuclear. pUL55 participated the assembly and maturation procedures of virus in some uncertain way. Characterization of pUL55 gave some insights of this gene and DEV investigation. However, further researches about this gene are expected to give more evidence in future.

## Competing interests

The authors declare that they have no competing interests.

## Authors' contributions

YW carried out most of the experiments and drafted the manuscript. ACC, MSW, SCZ, DKZ, RYJ, QHL, ZLC and XYC helped in experiments and drafted the manuscript. All authors read and approved the final manuscript.
